# Correlation of Xpert MTB/RIF with measures to assess *Mycobacterium tuberculosis* bacillary burden in high HIV burden areas of Southern Africa

**DOI:** 10.1038/s41598-018-23066-2

**Published:** 2018-03-26

**Authors:** Fenella Beynon, Grant Theron, Durval Respeito, Edson Mambuque, Belen Saavedra, Helder Bulo, Sergi Sanz, Keertan Dheda, Alberto L. Garcia-Basteiro

**Affiliations:** 1 ISGlobal, Hospital Clínic - Universitat de Barcelona, Barcelona, Spain; 2 DST/NRF Centre of Excellence for Biomedical Tuberculosis Research, MRC Centre for Molecular and Cellular Biology, Division of Molecular Biology and Human Genetics, Faculty of Medicine and Health Sciences, Stellenbosch University, Stellenbosch, South Africa; 3 Centro de Investigação em Saude de Manhiça (CISM). Rua 12, Cambeve CP 1929, Maputo, Mozambique; 4 Amsterdam Institute for Global Health and Development (AIGHD), Academic Medical Center, Amsterdam, The Netherlands

## Abstract

Traditionally, smear microscopy has been used as a point-of-care measure of bacillary burden in tuberculosis patients to inform infection control and contact tracing. Xpert MTB/RIF has the potential to replace smear. However, data to support the use of its quantitative output [cycle threshold (C_T_)] as an alternate point-of-care measure of bacillary burden are limited. This study assessed the correlation (Spearman’s) between C_T_, smear, culture time-to-positivity (TTP), and clinical factors in patients with Xpert-positive sputum from Mozambique (n = 238) and South Africa (n = 462). Mean CT and smear grade correlated well (ρ0.72); compared to TTP and smear (ρ0.61); and mean C_T_ and TTP (ρ0.50). In multivariate analyses, lower C_T_ (higher bacillary load) was associated with negative HIV serostatus and low BMI. A smear positivity rule-out (95% sensitivity) C_T_ cut-off of 28.0 was identified, with 54.1% specificity, 2.07 positive likelihood ratio, 0.09 negative likelihood ratio and 79.0% correctly classified. Cut-offs were higher for HIV positive compared to HIV negative individuals for any set sensitivity level. This study suggests Xpert C_T_ values correlate well with smear, both in HIV positive and negative individuals, and that C_T_ cut-offs might be broadly applicable to multiple settings. Studies to directly assess the association of C_T_ with infectiousness are needed.

## Introduction

The bacillary burden of tuberculosis (TB) in sputum is a key marker of transmission risk and disease severity^1–5^. Until recently, smear microscopy has been the mainstay of rapid TB diagnosis but lacks sensitivity, particularly in people living with HIV. Smear status has been used as a marker of transmission risk for decades, on the basis that smear positive patients are more likely to be infectious, particularly at the highest grade^6,7^.

Culture remains the gold standard for TB diagnosis. Liquid culture using the mycobacterial growth indicator tube (MGIT) system (BACTEC) has a high sensitivity with the ability to detect down to 1 colony forming unit (CFU) per sample, using solid culture as a reference standard^8^. Time to positivity (TTP) of the MGIT system (the amount of time in culture until mycobacterium tuberculosis (MTB) is detected) has been shown to predict the risk of transmission^9^ and response to treatment^10,11^ more reliably than smear status. However, in low-income countries culture is often not available due to its high cost and requirement for strict biohazard precautions^12^. Moreover, TTP cannot be used to aid decision-making about transmission curbing as it takes days to weeks to obtain results.

In 2010, the World Health Organisation (WHO) endorsed the roll-out of Xpert® MTB/RIF^13^, a cartridge based automated polymerase chain reaction (PCR) system for the detection of MTB deoxyribonucleic acid (DNA). Xpert provides results in under 2 hours and can detect down to 100 CFU per sample^8^, improving the sensitivity for patients with low bacillary burden compared to smear microscopy^14^. It also provides a cycle threshold (C_T_) result, which reflects the number of PCR cycles required to detect MTB. Each subsequent cycle represents ~50% less starting material than the last, thereby providing a semi-quantitative result of bacillary burden, with higher C_T_ results reflecting lower bacillary burden.

In-line with the WHO recommendation, 15 countries in one of the high-burden lists (TB, TB-HIV or MDR-TB) have now adopted Xpert as the initial diagnostic test for TB^15^. In these settings, there is a need for an alternative measure of bacillary burden that correlates with smear positivity to help predict transmission risk and disease severity. Xpert C_T_ could potentially be used as such a measure until evidence is available that directly assesses the relationship between baseline Xpert C_T_ and outcomes.

A small number of studies have demonstrated a correlation between TTP and C_T_ results in pulmonary samples, suggesting that an assessment of bacillary burden can be made that is more precise than smear microscopy and cheaper and faster than culture^8,16–19^. However, Hanrahan *et al*. demonstrated that the correlation of TTP and C_T_ is less strong among HIV positive individuals comparted to HIV negative individuals^18^. A recent meta-analysis identified cut-of C_T_ values of 27.7 and 31.8 to predict smear positivity with 85% and 95% sensitivity respectively^20^.

In this study, we aimed to further evaluate the correlation between measures of bacillary burden and identify a cut-point for smear positivity using Xpert C_T_. We have done so through the inclusion of several study sites in Mozambique and South Africa in the context of a high HIV prevalence with a larger sample size than previous studies. Furthermore, we evaluated the effect of clinical features and symptomatology on the different measures of bacillary burden.

## Methods

### Study sites and design

The analysis was conducted on anonymised data from participants of larger TB studies conducted in Mozambique and South Africa with sputum samples positive for Xpert MTB/RIF. In Mozambique, consecutive individuals aged 15 or over presenting with possible TB to any of the 10 clinics in the catchment area of Manhiça District Hospital were recruited from August 2013 to August 2014. Manhiça District Hospital is located in a high HIV and TB burden area in Maputo Province, in the south of the country^21^. In South Africa, data from six studies that recruited consecutive patients with presumptive TB from primary care clinics or hospitals were included, four of which have been previously published^17,22–24^. While the district of Manhiça is a semi-rural area in Mozambique, the studies based in South Africa were conducted in the Cape Town metropole, yet both sites are in areas with high TB and HIV prevalence^21,25^.

Sociodemographic and clinical details including HIV status and CD4 count were collected for all participants with TB from standardised questionnaires at the time of diagnosis and clinical records. HIV testing was offered to those without a known result within the last three months.

Informed consent was obtained for all participants. The Mozambique study was approved by the CISM Scientific Committee, the CISM Institutional Review Board and the National Bioethics Committee of Mozambique; the South African studies were approved by the University of Cape Town Faculty of Health Sciences Ethics Committee and the City of Cape Town. All study methods were carried out in accordance with the relevant guidelines and regulations established by the National Bioethics Committees in Mozambique and South Africa.

### Data availability statement

The datasets generated during the current study are kept at the data center of CISM. An anonymized version of the dataset can be made available upon request to CISM’s Internal Scientific Committee (Email: cci@manhica.net).

### Diagnostic tests and measures of bacillary burden

All participants in Mozambique had two samples taken, which were pooled and centrifuged and tested for both smear microscopy (Ziehl Neelsen) and Xpert (MTB/RIF G4 cartridge Cepheid, Sunnyvale, CA). All participants in South Africa had at least one (maximum 3) sample tested using fluorescence microscopy and Xpert. If more than one smear result was available, the highest smear grade was used. Smear microscopy results were graded using the International Union Against Tuberculosis and Lung Disease standard^26^. The mean C_T_ (of positive probes A-E) was recorded for those with positive Xpert results. Liquid culture (BACTEC MGIT 960, BD Diagnostics, Franklin Lakes, NJ) was done, after decontamination with NALC-NaOH, only for those with a positive Xpert result and/or those who had been treated for TB in the past (for a minimum of 4 weeks). TTP (from starting the culture to positive results) was recorded in days.

The sample for this study included only patients with pulmonary samples who had positive Xpert results and an available smear result. Those with rifampicin resistance were excluded as the C_T_ of the probe(s) detecting resistance have values ≥ 4 C_T_ from any other^27^, which could skew the mean C_T_ value of the probes. Those with a C_T_ ≥ 34 for the internal positive control (IPC) – a measure of the presence of inhibitors – were also excluded as this has previously been shown to affect the quantitative ability of Xpert^19^.

### Data analysis

Data was analysed using Stata 13.1 (StataCorp, College Station, Texas, USA). Descriptive analysis was carried out and differences were assessed between study sites and according to HIV status. The distributions of C_T_ and TTP values were assessed and, as they did not follow a normal distribution, a nonparametric equality of medians test was used to assess differences by HIV status, CD4 count and other clinical factors including symptoms. Differences in smear positivity were assessed using chi-squared. Correlation between TTP, C_T_ and smear grade were assessed using Spearman’s ρ with 95% confidence intervals for all participants and by study site and HIV status. In case no statistical differences in the correlation of Xpert C_T_ and smear grade or TTP were observed, estimates for the optimal cut off for smear positivity could be estimated with data from both sites.

Optimum cut-off for smear positivity (considering as all smears scanty to 3+ as positive) was calculated using a receiver operating characteristic (ROC) curve. The cut-off to detect smear positivity with 99%, 95%, 90% and 85% sensitivity (for use as a ‘rule-out’ cut-off) were derived from the combined data set and evaluated for sensitivity, specificity, negative and positive likelihood ratios and percentage of tests correctly classified overall and according to HIV status. Differing levels of C_T_ cut-offs were also assessed for sensitivity, specificity, negative and positive likelihood ratios and percentage of tests correctly classified overall and according to HIV status. Rule-in C_T_ cut-offs (95% specificity for smear positivity) were not calculated due to their poor clinical utility – previous studies have demonstrated a high degree of misclassification of smear positives as smear negative^28^. Univariate regression analysis was conducted to identify variables associated with measures of bacillary burden (age, sex, type of sample, history of prior treatment, HIV status, CD4 count, anti-retroviral therapy (ART), body mass index (BMI), presence and duration of constitutional symptoms or cough) and study site. Multivariate regression analysis was conducted including variables with p < 0.10 from univariate analysis. Bidirectional stepwise multivariate regression analysis was also conducted to ensure the validity of this approach.

## Results

### Baseline characteristics

Seven hundred Xpert positive participants were included – 238 (34.0%) from Mozambique and 462 from South Africa. Participant characteristics according to each site are shown in Table 1. Median age was 35 years (IQR 28–44) and 404 (58.1%) were men. A prior history of TB treatment was found in 29.1% of patients. Co-infection with HIV was common (57.2%) with significant immunosuppression – median CD4 was 173 (IQR 69–336).

**Table 1:** Participant characteristics by study site and overall (n = 700).

	Study site		Total
	Mozambique	South Africa	
	n = 238 n (%)/median (IQR)	n = 462 n(%)/median(IQR)	n = 700
Age	35.0 (29–46)	35.6 (28–44)	35.3 (28–45)
Sex			
Female	101 (42.4)	191 (41.7)	292 (42.0)
Male	138 (57.6)	267 (58.3)	404 (58.1)
Previous TB			
No	207 (87.0)	289 (62.6)	496 (70.9)
Yes	31 (13.0)	173 (37.5)	204 (29.1)
HIV			
Negative	58 (24.7)	235 (52.3)	293 (42.8)
Positive	177 (75.3)	214 (47.7)	391 (57.2)
CD4			
<200	85 (56.7)	118 (58.1)	203 (57.5)
≥200	65 (43.3)	85 (41.9)	150 (42.5)
On ART			
No	88 (57.9)	122 (70.1)	210 (64.4)
Yes	64 (42.1)	52 (29.9)	116 (35.6)
Smear Grade			
Negative	91 (38.2)	127 (28.0)	218 (31.6)
Scanty	7 (2.9)	63 (13.9)	70 (10.1)
1+	34 (14.3)	85 (18.8)	119 (17.2)
2+	45 (18.9)	78 (17.2)	123 (17.8)
3+	61 (25.6)	100 (22.1)	161 (23.3)
MGIT			
Negative	9 (3.8)	38 (8.3)	47 (6.8)
Positive	207 (87.0)	420 (91.7)	627 (90.1)
Contaminated	22 (9.2)	0	22 (3.2)
TTP	7.5 (5.3–11.6)	12.0 (7.0–16.0)	10 (7–15)
Mean C_T_	20.1 (15.7–26.6)	21.6 (17.6–26.8)	21.2 (17.0–26.8)

Symptoms were common: cough was present in 614/697 (88.1%) participants with documented history; fever in 347/592 (58.6%); weight loss in 609/700 (87.0%); and night sweats in 574/700 (82.0%). There were significant variations in symptomatology between study sites (Supplementary Table S1**)**. There were also differences between HIV positive and negative participants. Participants with HIV coinfection were more likely to have productive cough (85.0% vs 76.6%, p 0.010); weight loss (90.5% vs 82.9%, p 0.003); and fatigue (73.7% vs 61.1, p < 0.001); but less likely to have chest pain (45.5% vs 62.3%, p < 0.001).

### Measures of bacillary burden

Sputum smear was positive in 68.4% of participants; a greater proportion were smear positive in the South Africa cohort (72.0%) than the Mozambique cohort (61.8%), p < 0.001. Culture was positive in 90.1% of cases with a median TTP of 10 (IQR 7–15), which was significantly shorter in the Mozambique cohort than the South African – 7.5 (5.3–11.6) vs. 12.0 (7.0–16.0), p < 0.001. Mean C_T_ was slightly lower in the Mozambique cohort – 20.1 (15.7–26.6) vs 21.6 (17.6–26.8), p 0.016.

### Factors affecting bacillary burden

Factors associated with bacillary burden varied according to the individual measure **(**Supplementary Table S2). In univariate regression analysis only age, HIV and BMI were significantly associated with smear status, but symptoms of cough, productive cough, fever, weight loss, fatigue and chest pain as well as HIV, CD4, age, a prior history of TB and low BMI were variably associated with C_T_ and TTP. In multivariate regression analyses (Table 2), lower bacillary burden (higher mean C_T_, higher TTP and smear negativity) was associated with positive HIV status: mean C_T_ – B coefficient 2.59 [95% CI 1.58–3.59]; TTP – B coefficient 1.72 [0.33 to 3.11]; smear – OR 0.46 [95% CI 0.31–0.68]. Mean C_T_ was also associated with fever – B coefficient −1.27 [95% CI −2.28 to −0.27], cough – B coefficient −4.50 [−7.84 to −1.17] and BMI – B coefficient 2.23 [1.20 to 3.25] i.e. higher bacillary burden was associated with the presence of fever and cough and BMI of <18.5. TTP was also associated with study site – B coefficient 4.73 [3.32–6.13]. Smear was also associated with BMI – OR 0.63 [95% CI 0.43 to 0.92] and study site OR 1.54 [1.06 to 2.24].

**Table 2:** Multivariate regression analysis of factors significantly associated with higher sputum bacillary burden according to mean C_T_ of Xpert MTB/RIF, MGIT TTP and sputum smear microscopy.

	Mean C_T_				TTP				Smear positivity			
	Univariate analysis		Multivariate analysis		Univariate analysis		Multivariate analysis		Univariate analysis		Multivariate analysis	
	Β coefficient (95% CI)	p	Β coefficient (95% CI)	p	Β coefficient (95% CI)	p	Β coefficient (95% CI)	p	Odds Ratio (95% CI)	p	Odds Ratio (95% CI)	p
**HIV**												
Negative	Ref		Ref		Ref		Ref		Ref		Ref	
Positive	2.4 (1.46 to 3.33)	**<0.001**	2.59 (1.58 to 3.59)	**<0.001**	1.59 (0.35 to 2.82)	**0.011**	1.72 (0.33 to 3.11)	**0.015**	0.39 (0.28 to 0.56)	**<0.001**	0.46 (0.31 to 0.68)	**<0.001**
**Fever**												
No	Ref		Ref		Ref				Ref			
Yes	−1.35 (−2.36 to −0.33)	**0.01**	−1.27 (−2.28 to −0.27)	**0.013**	−2.79 (−4.15 to −1.43)	**<0.001**			1.18 (0.83 to 1.66)	0.36		
**Cough**												
No	Ref		Ref		Ref				Ref			
Yes	−3.29 (−4.72 to −1.87)	**<0.001**	−4.5 (−7.84 to −1.17)	**0.008**	1.84 (−0.04 to 3.73)	0.06			0.67 (0.40 to 1.14)	0.14		
**BMI**												
<18.5	Ref		Ref		Ref				Ref		Ref	
≥18.5	2.24 (1.19 to 3.28)	**<0.001**	2.23 (1.20 to 3.25)	**<0.001**	2.14 (0.72 to 3.57)	**0.003**			0.69 (0.47 to 1.00)	**0.048**	0.63 (0.43 to 0.92)	**0.019**
**Study site**												
Moz	Ref				Ref		Ref		Ref		Ref	
SA	1.1 (0.12 to 2.08)	**0.028**	(−0.89 to 1.38)		3.37 (2.11 to 4.17)	**<0.001**	4.73 (3.32 to 6.13)	**<0.001**	3.37 (2.11 to 4.17)	**<0.001**	1.54 (1.06 to 2.24)	**0.024**

### Correlation of measures of bacillary burden

**Figure 1:**
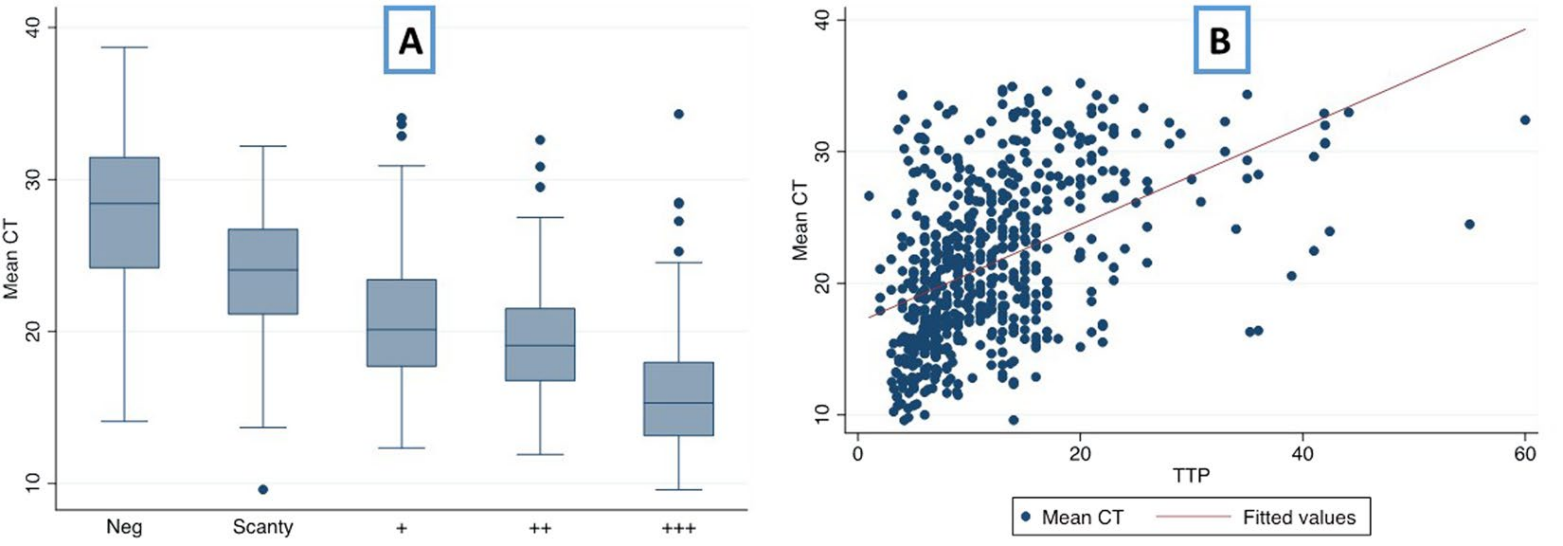
(**A**) Boxplot of mean cycle threshold (C_T_) – (average of positive Xpert MTB/RIF probes) according to smear grade and (**B**) Correlation between mean cycle threshold (C_T_) and time to culture positivity (TTP) in days.

Figure 1 demonstrates the variation of mean C_T_ according to smear grade and correlation of mean C_T_ with TTP. Smear negative samples had a median C_T_ of 28.4 (IQR 24.2–31.44) compared to 18.7 (15.8–22.6) for smear positive samples. Mean C_T_ and smear grade (n = 700) were the most closely correlated measures of bacillary burden (Spearman’s ρ 0.72 (95% CI 0.68–0.76); Mozambique ρ 0.76 (0.71–0.81); South Africa ρ 0.71 (0.66–0.75)). TTP correlated with smear grade (n = 620) with a Spearman’s ρ of 0.61 (0.56–0.66) (Mozambique 0.56 (0.45–0.64); South Africa 0.70 (0.65–0.75)). Mean C_T_ and TTP (n = 620) correlated least well with a ρ of 0.50 (0.44–0.56) (Mozambique 0.60 (0.51–0.68); South Africa 0.44 (0.36–0.52). Correlation varied by HIV co-infection – for mean C_T_ and smear grade, Spearman’s ρ were 0.64 (0.57–0.70) for HIV negative participants and 0.74 (0.69–0.78) for HIV positive participants; TTP and smear grade Spearman’s ρ 0.63 (0.55–0.69) and 0.58 (0.51–0.65); and mean C_T_ and TTP Spearman’s ρ 0.37 (0.26–0.47) and 0.56 (0.48–0.63) for HIV negative and positive respectively (Table 3).

**Table 3:** Correlation of measures of bacillary burden (Spearman’s ρ) with 95% confidence intervals for all samples and according to study site and HIV status.

	Overall	Mozambique	South Africa	HIV negative	HIV positive
	* n = 700	* n = 238	* n = 462	* n = 293	* n = 391
	§ n = 620	§ n = 207	§ n = 413	§ n = 266	§ n = 339
	ρ (95% CI)	ρ (95% CI)	ρ (95% CI)	ρ (95% CI)	ρ (95% CI)
Mean CT & smear (n = *)	0.72 (0.68–0.76)	0.76 (0.71–0.81)	0.71 (0.66–0.75)	0.64 (0.57–0.70)	0.74 (0.69–0.78)
TTP & smear (n = §)	0.61 (0.56–0.66)	0.56 (0.45–0.64)	0.70 (0.65–0.75)	0.63 (0.55–0.69)	0.58 (0.51–0.65)
Mean CT & TTP (n = §)	0.50 (0.44–0.56)	0.60 (0.51–0.68)	0.44 (0.36–0.52)	0.37 (0.26–0.47)	0.56 (0.48–0.63)

### CT cut-off for smear positivity

**Figure 2:**
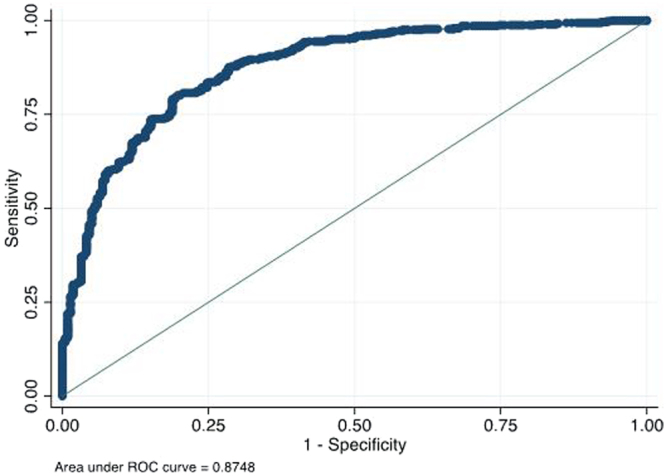
ROC curve analysis showing sensitivity for differing mean cycle threshold (C_T_) – (average of positive Xpert MTB/RIF probes) values as a test for smear positivity.

Figure 2 shows the ROC curve analysis of C_T_ threshold for smear positivity for overall data (area under the curve of 0.87). From the ROC curve analysis, a C_T_ cut-off value of 28.0 was identified with 95% sensitivity for smear positivity. Analysis according to HIV status found a lower cut-off for HIV negative individuals and higher cut-off for HIV positive individuals. Cut-offs with differing levels of sensitivity are reported in Table 4 (overall and according to HIV status) with associated, specificity, positive and negative likelihood ratios and percentage of correctly classified (with positive and negative predictive values in Supplementary Table S3. These are also presented according to different CT cut-offs between 25 and 30 in Table 5 and Supplementary Table S4. Cut-offs of 31.8 and 27.7 (sensitivity of 95%, specificity of 35% and sensitivity 85%, specificity 67%) identified by the recent meta-analysis of C_T_ predictors of smear positivity^20^, resulted in sensitivity of 98.8%, specificity 22.0% with a cut-off of 31.8 and sensitivity of 94.2% and specificity of 58.3% in this study.

**Table 4:** Cut-points of mean C_T_ to rule-out smear positivity (with varying degrees of sensitivity).

	C_T_ cut-off	Sensitivity % (95% CI)	Specificity % (95% CI)	Positive LR (95% CI)	Negative LR (95% CI)	Correctly classified % (95% CI)
**Cut-off for 99% sensitivity**						
Overall (n = 700)	32.6	99.0 (97.6–99.7)	15.6 (11.1–21.1)	1.17 (1.11–1.24)	0.07 (0.03–0.17)	73.0 (69.6–79.3)
HIV negative (n = 293)	29.6	99.1 (96.9–99.9)	31.7 (20.3–45.0)	1.45 (1.22–17.2)	0.03 (0.01–0.11)	85.3 (80.8–89.2)
HIV positive (n = 391)	32.9	99.2 (97.0–99.9)	11.6 (7.0–17.7)	1.12 (1.06–1.19)	0.07 (0.02–0.31)	64.5 (59.5–69.2)
**Cut-off for 95% sensitivity**						
Overall	28.0	95.0 (92.7–96.8)	54.1 (47.3–60.9)	2.07 (1.79–2.40)	0.09 (0.06–0.14)	82.3 (79.3–85.0)
HIV negative (n = 293)	27.3	95.3 (91.7–97.6)	48.3 (35.2–61.6)	1.84 (1.44–2.36)	0.10 (0.05–0.18)	85.7 (81.1–89.5)
HIV positive (n = 391)	29.2	95.3 (91.8–97.7)	47.7 (39.7–55.9)	1.82 (1.57–2.13)	0.10 (0.05–0.18)	76.5 (72.0–80.6)
**Cut-off for 90% sensitivity**						
Overall	26.3	90.3 (87.2–92.8)	65.1 (58.4–71.5)	2.59 (2.15–3.11)	0.15 (0.11–0.20)	82.4 (79.4–85.2)
HIV negative (n = 293)	25.2	90.1 (85.6–93.6)	66.7 (53.3–78.3)	2.70 (1.89–3.88)	0.15 (0.10–0.23)	85.3 (80.8–89.2)
HIV positive (n = 391)	27.1	90.3 (85.7–93.7)	64.5 (56.4–72.0)	2.54 (2.05–3.16)	0.15 (0.10–0.23)	80.1 (75.7–83.9)
**Cut-off for 85% sensitivity**						
Overall	24.6	85.1 (81.6–88.1)	72.0 (65.6–77.9)	3.04 (2.45–3.77)	0.21 (0.17–0.26)	81.0 (77.9–83.8)
HIV negative (n = 293)	23.5	85.0 (79.7–89.3)	73.3 (60.3–83.9)	3.18 (2.09–4.86)	0.20 (0.15–0.29)	82.6 (77.8–86.8)
HIV positive (n = 391)	25.6	85.6 (80.5–89.8)	71.6 (63.8–78.6)	3.01 (2.34–3.89)	0.20 (0.15–0.28)	80.1 (75.7–83.9)

Sensitivity, specificity, positive likelihood ratio (LR), negative LR and percentage of results correctly classified reported according to overall results (n = 700), HIV negative participants (n = 293) and HIV positive participants (n = 391).

**Table 5:** Sensitivity, specificity, positive likelihood ratio (LR), negative LR and percentage of results correctly classified reported according to different cut-points of mean C_T_ by overall results (n = 700), HIV negative participants (n = 293) and HIV positive participants (n = 391).

	C_T_ cut-off	Sensitivity % (95% CI)	Specificity % (95% CI)	Positive LR % (95% CI)	Negative LR % (95% CI)	Correctly classified %(95% CI)
**Overall**	25	86.5 (83.1–89.4)	71.6 (65.1–77.5)	3.05 (2.46–3.77)	0.19 (0.15–0.24)	81.9 (78.8–84.6)
HIV negative		89.7 (85.1–93.3)	66.7 (53.3–78.3)	2.69 (1.88–3.86)	0.15 (0.10–0.23)	85.0 (80.4–88.9)
HIV positive		83.5 (78.1–88.0)	72.9 (65.2–79.7)	3.08 (2.36–4.01)	0.23 (0.17–0.31)	79.3 (74.9–83.2)
**Overall**	26	89.2 (86.1–91.8)	68.8 (62.2–74.9)	2.86 (2.34–3.49)	0.16 (0.12–0.21)	82.9 (79.9–85.6)
HIV negative		91.4 (87.1–94.7)	61.7 (48.2–73.9)	2.39 (1.73–3.30)	0.14 (0.09–0.22)	85.3 (80.8–89.2)
HIV positive		86.9 (81.9–90.9)	71.0 (63.1–78.0)	3.00 (2.33–3.85)	0.18 (0.13–0.26)	80.6 (76.3–84.4)
**Overall**	27	92.1 (89.3–94.4)	60.6 (53.7–67.1)	2.34 (1.98–2.76)	0.13 (0.09–0.18)	82.3 (79.3–85.0)
HIV negative		94.0 (90.1–96.7)	48.3 (35.2–61.6)	1.82 (1.42–2.33)	0.12 (0.07–0.22)	84.6 (80.0–88.6)
HIV positive		89.8 (85.3–93.4)	64.5 (56.4–72.0)	2.53 (2.04–3.14)	0.16 (0.11–0.23)	79.8 (75.5–83.7)
**Overall**	28	95.0 (92.7–96.8)	54.1 (47.3–60.9)	2.07 (1.79–2.40)	0.09 (0.06–0.14)	82.3 (79.3–85.0)
HIV negative		97.0 (93.9–98.8)	40.0 (27.6–53.5)	1.62 (1.31–1.99)	0.08 (0.03–0.17)	85.3 (80.8–89.2)
HIV positive		92.8 (88.7–95.8)	59.4 (51.2–67.2)	2.28 (1.88–2.77)	0.12 (0.08–0.20)	79.5 (75.2–83.4)
**Overall**	29	96.5 (94.4–97.9)	45.9 (39.1–52.7)	1.78 (1.58–2.02)	0.08 (0.05–0.13)	80.7 (77.6–83.6)
HIV negative		97.9 (95.1–99.3)	35.0 (23.1–48.4)	1.51 (1.25–1.81)	0.06 (0.02–0.16)	85.0 (80.4–88.9)
HIV positive		94.9 (91.3–97.4)	49.7 (41.6–57.8)	1.89 (1.61–2.21)	0.10 (0.06–0.18)	77.0 (72.5–81.1)
**Overall**	30	97.5 (95.7–98.9)	39.9 (33.4–46.7)	1.62 (1.45–1.81)	0.06 (0.03–0.11)	79.6 (76.4–82.5)
HIV negative		99.1 (96.9–99.9)	28.3 (17.5–41.4)	1.38 (1.18–1.62)	0.03 (0.01–0.13)	84.6 (80.0–88.6)
HIV positive		95.8 (92.4–98.0)	43.9 (35.9–52.1)	1.71 (1.48–1.97)	0.10 (0.05–0.18)	75.2 (70.6–79.4)

## Discussion

This study has added to the evidence that Xpert MTB/RIF C_T_ can be used as a measure of bacillary burden that correlates well with smear grade and moderately with TTP in liquid culture. Given the current recommendation to replace smear with Xpert as the initial diagnostic test for TB and the lack of access to (and delay in obtaining) results from culture in LICs, Xpert may provide the only means for assessing bacillary burden for many countries.

This study identified a cut-off of 28 to rule-out smear positivity from studies in high HIV burden settings in Southern Africa. In studies reported by Hanrahan *et al*.^18^, Theron *et al*.^28,29^ and meta-analysis by Lange *et al*.^20^, variation of C_T_ values occurs within smear grades and the C_T_ cut-point to rule-out smear positivity varied from 27.7 to 31.8. This may be attributable to a number of factors such as variation in HIV prevalence and differences in type of smear microscopy performed. The sensitivity of any particular cut-off value of mean C_T_ varied according to HIV status, with lower sensitivity for HIV positive individuals. This may be due to the lower bacillary burden in HIV positive individuals as reflected by higher rates of smear negativity, and higher mean C_T_ and TTP values.

Whilst the majority of transmission occurs from individuals with higher smear grades, on which the basis of isolation and contact tracing for smear positive cases has been founded, some transmission does occur from smear negative individuals^30,31^. Factors contributing to this include intensity and duration of exposure, host susceptibility and discordance between sputum and cough aerosol bacillary burden^32^. Variation within the broad categorization of smear grades may also contribute to this and a more precise measure of bacillary burden by Xpert C_T_ could therefore prove useful in more accurately identifying those with the highest risk of transmission at the time of diagnosis to prioritise infection control measures. Until that time, rule-out C_T_ cut-offs have a greater utility than rule-in tests from a public health perspective: while rule-in cut-offs have a high specificity, they have poor sensitivity and therefore misclassify potentially infectious smear positive cases as smear negative^28^.

Baseline TTP is a better determinant than smear status of the risk of transmission^9^ and response to treatment^4,10,11^ but its cost and need for specialist laboratories and training are prohibitive and it is therefore used infrequently in many settings. Exemplifying this is the fact that among the 22 high TB burden countries, there are only 1.8 laboratories able to perform TB culture per 5 million population^12^. Using C_T_ as a same-day measure of bacillary burden that correlates with TTP could provide a useful and more timely surrogate. Unfortunately, C_T_ has been found to have variable correlation with TTP. In some studies, this correlation has been adversely affected by HIV coinfection^18^, likely due to the lower bacillary burden in these individuals. In this study, the correlation between C_T_ and TTP was stronger in HIV positive compared to HIV negative individuals. This could possibly be due to a difference in viable bacilli according to immunosuppression, with potentially a greater number of live bacilli in HIV positive individuals. C_T_ likely correlates more closely with smear given that both methods detect both viable and non-viable organisms compared to culture which only detects viable organisms. This is reflected in the finding that Xpert remains positive beyond culture conversion^33^ and patients with previously treated TB are more likely to have Xpert-positive, culture-negative results^34^. Ultimately, studies to directly assess outcomes and transmission based on CT results would be helpful in understanding its utility in clinical practice.

In seeking to develop understanding of the effect of different clinical factors on bacillary burden by these different methods, this study found that many symptoms of TB were associated with higher bacillary burden, however many of their effects on bacillary burden were not present in multivariate analysis suggesting that other factors contribute to this association, including HIV. In multivariate analysis, the only factors remaining significant for higher bacillary burden were HIV (negative), fever, cough, low BMI and study site. This likely reflects the increased severity of pulmonary disease and therefore a higher sputum bacillary load. Given that fever and BMI were associated with higher bacillary burden as measured by TTP, it may be valuable to consider them as clinical indicators of increased disease severity and transmission. Association of bacillary burden with study site may represent differences in the patient population not accounted for in the analysis (e.g. differences in disease severity or presence of pulmonary cavities. Although in general the diagnostic procedures were the same, there might still be subtle differences in laboratory processing techniques, samples chosen for culture/Xpert or different expertise of lab technologists involved which may also contribute to the findings. As Xpert mean C_T_ was not associated with study site, which may reflect the lower inter-operator variability compared to smear and TTP. Correlations between measures of bacillary burden may also be affected by calibration, laboratory processing techniques or differences in the frequency of non-culturable bacilli between populations due to factors leading to immune system containment such as HIV or TB treatment.

Beyond the potential differences by study site in the diagnostic procedures specified previously, this analysis had several limitations. Both sites have high HIV prevalence and are within Southern Africa, therefore it is possible that the findings are not applicable to other settings. Only patients with positive Xpert results were included; culture was not performed on all individuals in the larger study of patients with suspected TB, a reflection of real-world practice in this context. It was therefore not possible to evaluate the TTP for those who were Xpert negative, however these patients are likely to have lower bacillary burden and therefore present a lower transmission risk. However Xpert negative patients have a lower risk of adverse outcomes^35,36^. The study population therefore reflected a population with higher risk of adverse outcomes, in whom measurements of bacillary burden are likely to be more important.

Another limitation of the study was the exclusion of extra-pulmonary samples due to their very small number in the larger study population. Few studies to date have assessed this and found the correlation to be lower in extra-pulmonary samples, but further larger studies are warranted to assess whether C_T_ is reliable as a measure of bacillary burden in specific types of extrapulmonary sample^16,29^. In addition, transmission is almost exclusively from TB in sputum, reinforcing the greater need to understand bacillary burden in pulmonary samples.

With the introduction of Xpert Ultra recently recommended by WHO^37^, further studies are needed to evaluate the correlation of Xpert Ultra’s C_T_ with TTP and smear. However, whilst programmes continue to use Xpert MTB/RIF these findings remain applicable.

## Conclusion

This study suggests that Xpert C_T_ can be used as a measure of bacillary burden and a surrogate for smear status, with the recommended cut-off of 28 to rule-out smear positivity. However there is variation in the recommended cut-off between studies which may reflect differences in clinical features of the population. The exact cut-off point that individual national TB programmes that have replaced smear with Xpert will also depend on the specific desire for sensitivity and specificity. However, until studies have been conducted that directly assess the value of C_T_ with outcomes and transmission of TB, C_T_ values could be viewed as a measure of bacillary burden, with lower C_T_ values representing a higher transmission risk, for programmes that no longer utilise smear microscopy.

## Electronic supplementary material

Supplementary Tables
