# Supplementary material for: Correlation of Xpert MTB/RIF with measures to assess Mycobacterium tuberculosis bacillary burden in high HIV burden areas of Southern Africa
Source: Sci Rep. 2018 Mar 26;8:5201. doi: 10.1038/s41598-018-23066-2 (PMC5980110; doi:10.1038/s41598-018-23066-2)
Supplement: Supplementary file 1 — Supplementary Tables [file 41598_2018_23066_MOESM1_ESM.doc]

**Supplementary Information**

**Manuscript Title:**

Correlation of Xpert MTB/RIF with measures to assess *Mycobacterium tuberculosis* bacillary burden in high HIV burden areas of Southern Africa

**Authors:**

Fenella Beynon^1^, Grant Theron^2^, Durval Respeito^3^, Edson Mambuque^3^, Belen Saavedra^3^, Helder Bulo^3^, Sergi Sanz^1^, Keertan Dheda^2^, Alberto L. Garcia-Basteiro^1,3,4^

**Affiliations:**

^1^ISGlobal, Barcelona Ctr. Int. Health Res. (CRESIB), Hospital Clínic - Universitat de Barcelona, Rossello, 132, 08036, Barcelona, Spain.

^2^DST/NRF Centre of Excellence for Biomedical Tuberculosis Research, MRC Centre for Molecular and Cellular Biology, Division of Molecular Biology and Human Genetics, Faculty of Medicine and Health Sciences, Stellenbosch University.

^3^ Centro de Investigação em Saude de Manhiça (CISM). Rua 12, Cambeve CP 1929 Maputo, Mozambique.

^4^Amsterdam Institute for Global Health and Development (AIGHD), Academic Medical Center, Amsterdam, The Netherlands

**Corresponding author:**

*Alberto L. Garcia-Basteiro

Centro de Investigação em Saúde de Manhiça (CISM)
Tel.: (+258) +258 866 57 57 07 Email address: [alberto.garcia-basteiro@manhica.net](mailto:alberto.garcia-basteiro@manhica.net)

**Supplementary Table S1.** Symptoms according to study site and overall

| **Symptom / sign** | **Mozambique**  **(n=238)**  n (%) | **South Africa**  **(n=462)**  n (%) | **Total**  **(n=294)**  n (%) |
| --- | --- | --- | --- |
| **Cough**  **> 14 Days** | 231 (97.1)  213 (92.6) | 383 (83.4)  314 (74.8) | 614 (88.1)  527 (81.1) |
| **Productive cough**  **> 14 Days** | 219 (92.0)  204 (88.7) | 270 (72.6)  244 (65.6) | 489 (80.2)  448 (74.4) |
| **Haemoptysis** | 16 (6.9) | 35 (8.5) | 51 (7.9) |
| **Fever** | 195 (81.9) | 152 (42.9) | 347 (58.6) |
| **Weight loss** | 215 (90.3) | 394 (85.3) | 609 (87.0) |
| **Night sweats** | 193 (81.1) | 381 (82.5) | 574 (82.0) |
| **Fatigue** | 170 (71.4) | 302 (65.4) | 472 (67.4) |
| **Dyspnoea** | 37 (15.6) | 228 (65.4) | 265 (44.8) |
| **Chest pain** | 76 (32.1) | 234 (66.1) | 310 (52.5) |
| **BMI <18.5** | 109 (46.6) | 101 (29.2) | 210 (36.2) |

**Supplementary Table S2.** Factors associated with bacillary burden across both sites according to mean CT of Xpert MTB/RIF, MGIT TTP and sputum smear microscopy

|  | **Mean C_T_**  **Median (IQR)** | **p**  **value** | **TTP**  **Median (IQR)** | **p**  **value** | **Smear positivity n (%)** | **p**  **value** |
| --- | --- | --- | --- | --- | --- | --- |
| **Age**  **≤35**  **>35** | 16.7 (20.4-25.7)  17.4 (22.1-28.0) | **0.007** | 6.1 (9-15)  7 (10.2-15) | 0.48 | 252 (72.8)  230 (65.0) | **0.025** |
| **PrevTB**  **No**  **Yes** | 16.9 (21.2-26.9)  17.2 (21.5-26.6) | 0.76 | 6.0 (9.0 -14.4)  7.0 (11.0-15.0) | **0.027** | 335 (67.5)  147 (72.1) | 0.24 |
| **HIV**  **Negative**  **Positive** | 16.3 (19.6-23.9)  17.9 (22.6-28.5) | **<0.001** | 6 (9-14)  7.0 (10.9 – 16) | **0.007** | 233 (79.5)  236 (60.4) | **<0.001** |
| **CD4**  **<200**  **≥200** | 18.5 (23.8-29.2)  17.2 (20.9-28.3) | **0.020** | 7.5 (12-17.3)  6.8 (10-15.4) | 0.08 | 121 (59.6)  96 (64.0) | 0.40 |
| **Cough**  **No**  **Yes** | 20.4 (24.2-29.6)  16.7 (20.6-26.3) | **<0.001** | 6 (8 – 12.1)  6.7 (10-15) | **0.022** | 63 (75.9)  417 (67.9) | 0.14 |
| **Productive**  **No**  **Yes** | 19.4 (23.3-29.2)  16.3 (20.3-26.2) | **<0.001** | 6 (8.8-13.0)  6.9 (10.2-15.1) | **0.045** | 86 (71.1)  334 (68.3) | 0.56 |
| **Fever**  **No**  **Yes** | 17.2 (21.6-27.1)  16.0 (19.9-26.0) | **0.011** | 8 (12-16)  6 (9-14) | **<0.001** | 160 (65.3)  239 (68.9) | 0.36 |
| **Weight loss**  **No**  **Yes** | 18.8 (22.4-28.0)  16.7 (20.9-26.6) | **0.024** | 6.8 (10-15)  6.4 (10-15) | 0.56 | 60 (65.9)  422 (69.3) | 0.52 |
| **Fatigue**  **No**  **Yes** | 17.3 (21.7-27.5)  16.7 (20.9-26.6) | 0.14 | 6 (9-14)  7.0 (10.2-15.1) | **0.027** | 166 (72.8)  316 (67.0) | 0.12 |
| **Chest pain**  **No**  **Yes** | 16.3 (20.5-26.6)  16.8 (20.5-26.1) | 0.69 | 6 (9-13.8)  7.5 (12-16) | **<0.001** | 183 (65.1)  215 (69.4) | 0.27 |
| **BMI**  **<18.5**  **≥18.5** | 15.4 (19.0-24.5)  17.3 (21.5-27.2) | **<0.001** | 6.0 (8.7-14)  7.5 (12-16) | **<0.001** | 153 (72.9)  240 (64.9) | **0.048** |

**Supplementary Table S3.** Cut-points of mean C_T_ to rule-out smear positivity (with varying degrees of sensitivity). Sensitivity, specificity, positive likelihood ratio (LR), negative LR, positive predictive value (PPV) and negative predictive value (NPV) and percentage of results correctly classified reported according to overall results (n=700), HIV negative participants (n=293) and HIV positive participants (n=391).

|  | C_T_ cut-off | Sensitivity (%) | Specificity (%) | Positive LR | Negative LR | PPV  (%) | NPV  (%) | Correctly classified (%) |
| --- | --- | --- | --- | --- | --- | --- | --- | --- |
| Cut-off for 99% sensitivity | | | | | | | | |
| Overall (n=700) | 32.6 | 99.0 | 15.6 | 1.17 | 0.07 | 72.2 | 87.2 | 73.0% |
| HIV negative (n=293) | 29.6 | 99.1 | 31.7 | 1.45 | 0.03 | 84.9 | 90.5 | 85.3% |
| HIV positive (n=391) | 32.9 | 99.2 | 11.6 | 1.12 | 0.07 | 63.1 | 90.0 | 64.5% |
| **Cut-off for 95% sensitivity** | | | | | | | | |
| **Overall** | **28.0** | **95.0** | **54.1** | **2.07** | **0.09** | **82.1** | **83.1** | **79.0** |
| **HIV negative (n=293)** | **27.3** | **95.3** | **48.3** | **1.84** | **0.10** | **87.8** | **72.5** | **85.7%** |
| **HIV positive (n=391)** | **29.2** | **95.3** | **47.7** | **1.82** | **0.10** | **73.5** | **87.1** | **76.5** |
| Cut-off for 90% sensitivity | | | | | | | | |
| Overall | 26.3 | 90.5 | 65.1 | 2.59 | 0.15 | 85.1 | 75.1 | 82.4% |
| HIV negative (n=293) | 25.2 | 90.1 | 66.7 | 2.71 | 0.15 | 91.3 | 63.5 | 85.3% |
| HIV positive (n=391) | 27.1 | 90.3 | 64.5 | 2.54 | 0.15 | 79.5 | 81.3 | 80.1% |
| Cut-off for 85% sensitivity | | | | | | | | |
| Overall | 24.6 | 85.1 | 72.0 | 3.04 | 0.21 | 87.1 | 68.6 | 81.3% |
| HIV negative (n=293) | 23.5 | 85.0 | 73.3 | 3.18 | 0.20 | 92.5 | 55.7 | 82.6% |
| HIV positive (n=391) | 25.6 | 85.6 | 71.6 | 3.01 | 0.20 | 82.1 | 76.6 | 80.1% |

**Supplementary Table S4.** Sensitivity, specificity, positive likelihood ratio (LR), negative LR, positive predictive value (PPV) and negative predictive value (NPV) and percentage of results correctly classified reported according to different cut-points of mean C_T_ by overall results (n=700), HIV negative participants (n=293) and HIV positive participants (n=391).

|  | C_T_ cut-off | Sensitivity (%) | Specificity  (%) | Positive LR | Negative LR | PPV  (%) | NPV  (%) | Correctly classified (%) |
| --- | --- | --- | --- | --- | --- | --- | --- | --- |
| Overall | 25 | 86.5 | 71.6 | 3.05 | 0.19 | 87.1 | 70.6 | 81.9% |
| HIV negative |  | 89.7 | 66.7 | 2.69 | 0.15 | 91.3 | 62.5 | 85.0% |
| HIV positive |  | 83.5 | 72.9 | 3.08 | 0.23 | 82.4 | 74.3 | 79.3% |
| Overall | 26 | 89.2 | 68.8 | 2.86 | 0.16 | 86.3 | 73.9 | 82.9% |
| HIV negative |  | 91.4 | 61.7 | 2.39 | 0.14 | 90.2 | 63.8 | 85.3% |
| HIV positive |  | 86.9 | 71.0 | 3.00 | 0.18 | 82.0 | 78.0 | 80.6% |
| Overall | 27 | 92.1 | 60.6 | 2.34 | 0.13 | 83.8 | 77.7 | 82.3% |
| HIV negative |  | 94.0 | 48.3 | 1.82 | 0.12 | 87.6 | 67.4 | 84.6% |
| HIV positive |  | 89.8 | 64.5 | 2.53 | 0.16 | 79.4 | 80.7 | 79.8% |
| Overall | 28 | 95.0 | 54.1 | 2.07 | 0.09 | 82.1 | 83.1 | 79.0% |
| HIV negative |  | 97.0 | 40.0 | 1.62 | 0.08 | 86.3 | 77.4 | 85.3% |
| HIV positive |  | 92.8 | 52.4 | 1.95 | 0.14 | 77.7 | 84.4 | 79.5% |
| Overall | 29 | 96.5 | 45.9 | 1.78 | 0.08 | 79.9 | 85.6 | 80.7% |
| HIV negative |  | 97.9 | 35 | 1.51 | 0.06 | 85.7 | 81.5 | 85.0% |
| HIV positive |  | 94.9 | 49.7 | 1.89 | 0.10 | 74.2 | 86.5 | 77.0% |
| Overall | 30 | 97.5 | 39.9 | 1.62 | 0.06 | 78.2 | 87.9 | 79.6% |
| HIV negative |  | 99.1 | 28.3 | 1.38 | 0.03 | 84.3 | 89.5 | 84.6% |
| HIV positive |  | 95.8 | 43.9 | 1.71 | 0.10 | 72.2 | 87.2 | 75.2% |
